# The Professional Education of Dentists

**Published:** 1852-06

**Authors:** E. B. Gardette

**Affiliations:** Dentist


### *The Professional Education of Dentists.*

By E. B. Gardette, M. D., Dentist.

To the Editors of the Medical Examiner.

Gentlemen:—When I claimed the courtesy of you to republish my "Memorial" addressed to the Medical Colleges of our country, recommending the establishment of Lectures on Dental Surgery, I had no thought that the kindness you then extended would involve a necessity for asking to occupy another page of your valuable journal. I had no desire nor expectation to open an argument or controversy with other journals or individuals, as to the propriety or feasibility of the project then suggested solely from a wish for improvement in the mode of educating Dentists. Hence, I was careful to assail no one, but to make a sinple proposal, designedly in *memorial form*, for the consideration of the medical schools, who, if disposed to give it their attention, would necessarily seek information from all reliable sources before adopting any plan. Then would have been the time for the friends of such a scheme to defend it by argument, and consider the deficiencies felt to exist in other systems of professional education; and then too might its enemies properly make their objections.

But the Editor of the "Baltimore Journal of Dental Science," has seen fit, in a selfish fear lest the mere proposal itself should injure the prospects of the Baltimore Dental College (of which he is a professor), to misconstrue or misunderstand my language, and oppose the project of such Lectureships. That writer, in a determination to anticipate and forestall any action on the subject, pre-judges, passes sentence, and *would* hang up the proposition as an absurd abstraction, while at the same time he *professes* respect for, and attaches great importance to, the opinions of the proposer. It remains to be seen whether the sole and summary judgment of the Editor of the journal will sway those who are to determine this grave question.

My reliance upon the peaceful and polite character of my paper, did not save it from a headlong declaration of war by the Baltimore journal, an attack both inconsistent with the public parade of sentiments or professions towards me, and uncalled for or forbidden by its *memorial form* and careful expressions. My rejoinder in a few merely explanatory remarks, made through your journal in June last, did not prevent the Editor of the Journal of Dental Surgery, in his July number, from again intruding upon his readers the subject of my proposal and myself. On this occasion he is influenced by no higher considerations than before, at a moment when my private affairs did not permit me to see his remarks; and it is under the influences natural to this state of things, that I now speak of the Baltimore Journal. I wish to do so as one who discerns the springs of action that govern even so huge a machine—as a quarterly periodical. In this, my course is a defensive one, and due alike to those of my professional brethren who unite with me in contempt for the selfishness that will descend to quotations from parts of sentences, calculated to mislead the reader, as to the real statements that have been made the objects of its premature and unfair comments.

So far from having been "*very decided in my expressions of satisfaction at what I saw and heard at the Examinations of the Baltimore College of Dental Surgery*," it was, on the contrary, the dissatisfaction experienced then, which confirmed my previous opinion, that the establishment of dental colleges would not supply the want felt in reference to educating dentists, and would interfere with the eventual standing of the professson itself. It was this strengthened feeling, and none other, that finally induced the suggestion to have dental surgery taught, with all other specialities of medicine, in medical colleges. I am constrained to deny entirely having sanctioned the course of instruction in *Dentistry proper*, which I had the opportunity to see at the Baltimore College of Dental Surgery during two successive years, —1848 and 49; the observations and examinations then made, resulted in a permanent belief of its incapacity to fulfil the objects of its incorporation, or meet the hopes and wishes of educated, well bred dentists. I will not attempt to sustain the justice of this opinion by inquiries, that might be regarded as unkind or invidious, into practical effects upon the profession and the community, but the defective system through which young men have received half-way medical, and still less dental instruction, will prove itself and its influences in professional histories yet to be written.

In reference to my examination of *operations performed* by the pupils of the school, and my having approved of what I saw, which the Baltimore Professor states with a view to make my present opinions appear inconsistent or contradictory, the simple truth is that I saw nothing—for I did not see the operations *performed*; and it is too late in professional life with me, to determine the merits of a surgical operation, by looking at or examining its external surface. I could not know, of course, the previous condition of the teeth operated upon, the correct or incorrect exercise of judgment by which the young operators decided to form and fill cavities in the teeth with metal; I could not determine the propriety, in manner or extent, with which the sound or unsound parts had been removed; I could not judge of the time and skill and brains devoted to these manipulations, the good sense or professional theories exhibited in the application of knowledge, whether for determining upon or the performance of operations.

As regards the *artificial teeth* shown me on the occasion referred to, it is almost needless to say to Dentists that any smart goldsmith's apprentice, after a few months' experience, can get up such specimens at least as well, he having had no part in the most important duties of settling the extent or peculiarities of the artificial fixture, or its adaptation to living parts for the patient who must endure it in his mouth.

Neither the smooth and brightly burnished metal fillings or the specimens of plate work, not in use, exhibited as the performances of the graduates of Baltimore Dental College, could be regarded as evidences of successful teaching in either branch of professional education; they were looked at with kindly interest, but as a mere formality, and a natural disposition to encourage young gentlemen who had just graduated, (the only operations brought before me, were, as Chairman of a Committee of Examiners for awarding a prize to the best *graduate,*) would of course govern any expressions that could have been uttered on the occasion. The all important decision had been made; they were already admitted into the ranks of the profession. The number of competitors, to the best of my recollection, was eleven or twelve, and of these several had been practising dentists for several years before going to the College. The prize was awarded to a young gentleman who had been the industrions private pupil of a competent preceptor for two years, not so much for the show of operations, as for the modest intelligence and practical common sense he applied to his profession.

Such were the true impressions made upon my mind at the period to which the Editor of the Baltimore Journal has referred; they were never uttered until now, except confidentially to Dr. Hullihen, of Wheeling, and Dr. Harwood, of Boston, gentlemen as well as distinguished Dentists, whom I met at Baltimore. That I should have politely avoided the expression of such unwelcome views as those we entertained, to the faculty, of whom I was the kindly treated guest, is, I trust, only consistent with the ordinary good breeding which responds to attentions and hospitalities. The system of extending social civilities that shut the mouth for every opinion *unfavorable* to the objects of gentlemen who have extended them (I do not say *with* that object) is to be blamed for the want of frankness or plain dealing too common under such circumstances; it is open to the suspicion of belonging to the same tactics to which editorial life is subjected, in the gifts that *anticipate opinions,* and often purchase positive expressions favorable to the views of the giver, at the expense of facts and sincerity. But in either case, I am free to admit the impropriety of such a disregard of justice, and especially where serious professions, or institutions for obtaining knowledge, are concerned.

In proposing the establishment of professorships on dental surgery in medical colleges, I did not compare such a mode of educating dentists with that of existing dental colleges with which I was acquainted, simply because the comparison, as I conceived it, would have been no compliment to the latter; my advocacy of a better plan was at most a negative condemnation of the one pursued at Baltimore, and if I was mistaken in this preference, the result would have proved it so. For I believe that calling public attention to any new scheme of professional education for the dentist, even from a bad or impracticable one, some benefit would be likely to ensue, and especially to a superior existing system. But in truth the Baltimore Dental College had had a fair trial; it was, and is, unsuccessful; not, however, for the reasons assigned by the Editor of the Baltimore Journal, but really because it did not offer to the profession and the public materials that inspired confidence. With the single exception of Dr. H. H. Hayden, the College, it may be said without disparagement, has been the medium for lending reputation and importance to names but little known in the profession, rather than deriving strength and advantages from those who composed its dental faculty. The two chairs entrusted to medical men, I have reason to know, have been admirably filled, and these I am led to believe have sustained the College in its lingering existence; for although the "Professor of Principles of Practice" says that "*the Faculty have never yet raised the question among themselves, as to the individual importance of its members*," the profession, and even the pupils of the College, have very naturally and properly both "*raised the* question" and settled it too.

The Baltimore Journal quotes the polite expressions in Dr. Hullihen's Valedictory Address as good authority* for the usefulness or importance of the dental college. That address, I will merely say, was delivered at the invitation of the medical faculty, in their presence and that of their invited friends; and at a period when no other scheme for the education of dentists was under consideration; a passing compliment to the institution was but a civil return for that of inviting a gentleman unconnected with the College to become its organ in a valedictory to its pupils. The entire avoidance on *my part*, of any allusion, flattering or otherwise, to the school or its system, when placed under the same circumstances as Dr. Hullihen the year preceding, should,

*Not *very* good authority, when I recall the fact that its publication was suppressed, and it is the only Valedictory that ever was denied a place in the Baltimore Journal since the Dental College was commenced!

by the same rule and with equal propriety, be regarded as evidence that I did not approve of the scheme of dental colleges.

At any rate I am at liberty to say that Dr. Hullihen *is now, and has been from the first moment of my proposal, strongly in favor of establishing Professorships for Dental Surgery in Medical Colleges.*

The warm hearted Provost of the Baltimore College, Dr. E. Parmly, in his benevolent zeal for remedying the defects of dental education, has lent the benefit of his good name to aid that institution; but he has only visited it annually, at the period of its commencement, for the last five or six years, giving no time to the duties of instruction. Better evidence need not be sought in relation to his views of the education demanded for dentists, than the fact that while allowing his son and nephew (destined for dentists) to attend the Baltimore College of Dental Surgery, he caused the one to graduate an M. D. at the Washington Medical College of Baltimore, and is preparing the other for a similar degree in the anatomical schools of Paris.

We must look beyond the limited views of the Baltimore Journal, in order to determine the propriety and advantages, (not to *it*, but to the world) of incorporating the profession of dentist with its parent science of medicine; it is only thus that a correct ambition can be excited in men of the better and educated classes to join its ranks; it is only thus that the community will get the benefit of strong and cultivated minds applied to this branch of surgery.

It is but just to remark that the deficiencies I have been compelled reluctantly to refer to, as applicable to one Dental College, may not have equal force in reference to others that are differently organized, if there be such; but the main and general objection to these institutions, has seemed to me to be the sad consequences, present and prospective, that arise in the injurious separation of the dentist from the medical profession. It was in this spirit, and with regard to this serious objection, that my original proposition to have dental surgery taught in medical colleges, was published. I then made no needless reference to the inherent defects of the only Dental College with which I was acquainted.

It is no test of the two schemes proposed for dental education, to assert that better dentists will be made under the one or the other. This is not the true question, nor could it well be determined until both plans had been fairly tried. In either case, whether dental college, or dental professor in medical college, much will of course depend upon the talents and characters respectively, of pupils and teachers. But the real and important subject for consideration is, whether the dentist, being, or aspiring to be, a member of the medical profession, should not have, in education, a similar origin, and undergo the same preparation as the medical man, in addition to what he requires for his particular speciality. I make no concession in saying that a dental college with *good* teachers, would produce better dentists than a medical college with incompetent professors of dental surgery; and the converse of this proposition is equally true.

The New York Medical College has already under consideration the establishment of a chair for Dentistry; and the New York Journal of Medicine, for February, contains an able paper from the pen of Dr. John Trenor, a distinguished dentist of that city, in which he condemns dental colleges as "*so decidedly inefficient, that they are a greater drawback to improvement than if they had never existed. They profess to remedy an evil which they most effectually and glaringly magnify.*" The chief sense in which I understand and apply this sweeping condemnation from an experienced dentist and medically educated man, is in the effects produced, directly and indirectly, upon the advancement of the profession, as a branch of medicine, and upon the class of men by whom it is to be represented. For whatever may have been the defects or difficulties, heretofore existing, in educating dentists, the profession itself was still regarded as a speciality of surgery, and belonging to medicine; and however poorly it may have been represented, it had not been severed by distinct corporate powers from its parent science, by titles or otherwise, until the Baltimore College of Dental Surgery came into existence.

In connection with this view, I may appropriately quote from Lord Bacon, who, without practical knowledge, has looked at the same subjects from the illuminated heights of his abstract wisdom:

"And in particular sciences we see, that if men fall to subdivide their

"their labors, as to be an oculist in physic, or to be perfect in some one "title of the law or the like, they may prove ready and subtile, but not "deep or sufficient—no, not in that subject to which they do particularly "attend, because of that concert which it hath with the rest. . . . . I "mean not that use which one science hath of another for *ornament*, "but I mean it *directly* of that use by way of supply of light and in- "formation, which the particulars and instances of one science do yield "and present for the framing or correcting of the axioms of another "science in their very truth and notion; . . . . for sciences distin- "guished have a dependence upon universal knowledge, to be augmented "and rectified by the superior light thereof; as well as the parts and "members of a science have upon the maxims of the same science, and "the mutual light and consent which one part receiveth of another; " . . . . and so one science greatly aiding to the invention and aug- "mentation of another. And therefore, without this intercourse, the "axioms of science will fall out to be neither full nor true; but will be "such opinions as Aristotle in some places doth wisely censure, when "when he saith, 'These are the opinions of persons that have respect "but to a few things.'"

"*Few things*" have, I fear, narrowed down to dollars and cents, in the modern separation of the sciences, no less than in the conducting of some *scientific* periodicals, which instead of being devoted to the advancement of learning, are only anxious to make money and exercise "a little brief authority."

Professor Thomas E. Bond says, in his excellent work on Dental Medicine, "*An oculist, unless a thorough physician, would be utterly unqualified to treat diseases of the eye;*" and surely this rule scarcely applies more strictly to the oculist than to the dentist, especially when we have under consideration the best mode of educating either.

Prof. Bond also remarks in the same work, that "the practice of dental surgery was long degraded, from causes precisely similar to those evil influences which depressed similar branches of the art. Disregarded by educated men, it necessarily fell into the hands of the ignorant and rude; and precisely as surgery and midwifery have gradually emerged from their barbarous state and attendant disrepute, dentistry is now winning its way, against all opposition, to its proper consideration."

Now as dentistry has been degraded by the *same causes*, should not the evil be remedied by the *same means?* The specialities of surgery and midwifery were never *separated* from *general medicine*, *either* by *distinct* or *independent schemes* of *education*, or by *titles differing* from *those that* are *universal among medical men.* These specialities have reached the equality they enjoy with the other departments of the science *chiefly through the medium of professorships* in medical colleges devoted to them, and eventually through the character and general education of the men by whom they are represented.

Unfortunately I do not perceive that "dentistry *is* now winning its way to its proper consideration;" for although the number of its practitioners has greatly increased, with the sadly increasing need of them, those who best understand what really constitutes *good* dentists, seek in vain for the proof of the much talked of wonderful advance of dental surgery and dental literature. The London Medico-Chirurgical Journal of Practical Medicine,* noticing the pretensions of our noisy dental periodicals, has been severely and justly observant of the important distinction between true dental surgery, by which the health and preservation of the teeth are accomplished, and the modern mixture of mercantile and mechanical business in teeth, which characterises a great proportion of what is called dentistry in our country. The era in which we live has been prolific of improvement in the manufacture of teeth; but while this has been a valuable triumph in *honest* hands, for *art*, it has brought with it, I fear, a sad falling off, or increased difficulties to *science.* The readiness to sacrifice permanent utility to momentary show, is, perhaps, among the common errors of the times; it is a weakness natural to vanity and ignorance; and there really seems some danger, such is the almost universal display of white crockery in American mouths, both old and young, that we shall be nicknamed the nation of china-teeth. This comes from the facility and cheapness with which what are termed "*mechanical* dentists" supply sets of mineral teeth, and the great difficulty with which good surgeon-dentists are found, to preserve the natural teeth from disease. These causes seem to work like a two-edged sword, in destroying the luxury of sound and useful masticators. With so enormous a supply of mineral teeth, as are the subject of traffic in this country (the statistics are incredible), there must be a corresponding consumption; and the only convenient means of filling human mouths with such teeth, is to

* See Vol. 49, Am. repub.

empty them of the natural ones. The inference is painful; but I am disposed to think that the great increase in the demand for artificial teeth, or the extraordinary extent to which they are used, should be regarded as an evidence of the decline of dental surgery.

Unluckily we have other humbling proofs that our profession is not "winning its way against all opposition, to its proper consideration." We may see, for instance, in the leading journal of dental science, a *cold water cure* for *the exposed nerves* of teeth, from a correspondent who says he has discovered that the nerves of the human teeth are "*endowed with recuperative power.*" And from another favorite writer of the same journal we find a *serious recommendation of the use of a warming-pan for the gold foil, to regulate the temperature of the metal, before it is put into the cavity of a tooth!!!* This is *only* to be done in very cold weather, mind you, gentle reader.

The published discussions of certain dental societies, sometimes occupying nearly fifty pages of a periodical, may be referred to as painful-evidence of the present condition of dental surgery, and the unfortunate influences (among which I am forced to include dental colleges,) under which the profession of dentist is fast assuming, or re-assuming the unintellectual character of a trade. These things obtain the sanction and the sympathy of the editor of the Dental Journal, who at the same time frowns down a contributor of sensible and scientific matter, at variance with his doctrines, in which he presumes to tell the plain truth to ignorance, and who will not submit to the dictatorial requirements which the conductor of a public journal has no right to make.

These are among the evils that call for cure by a different and better system of education for the dentist, and which, I believe, will not be remedied until he is required to be a medical man, in the true sense of the term, identified, in the source and character of his knowledge, with the profession of medicine. Then, and not till then, may we hope to see that fellowship and free communion of thought, calculated to advance the science, between the members of the profession generally, and which is now clamored for by two distinct classes of dentists: first, by the artful and unworthy, who desire to benefit their business or borrow good character, which they will not earn; and next, by the amiable and philanthropic, who do not see into the designs of the cunning, and lend themselves, their means and reputations, thinking to advance the interests of the profession itself, by their intercourse with ignorant and unblushing pretenders. No such result, I need scarcely say, can be hoped for; and when the Journal argues that those who keep aloof from such association, are ashamed of their profession, or deem the name of dentist a reproach, he should rather infer that they only blushed for its representatives—men of "modest assurance," who open their offices, after little or no legitimate preparation, and at once call upon all in "*the same line of business*," with no other introduction than their own desire for "*professional reciprocity*"!

I cannot regard these general remarks, though incidental, as digressive from the question of dental education, or the scheme of Professorships in medical colleges, for its elevation and advancement. The gloss and deceptions thrown over the real character and condition of the dentist in this country, by a parade of trashy matter under empty titles, is the lamentable result of recent and present combinations, which have been ostensibly for the *improvement* of dental surgery, and it has, perhaps, derived a little unmeaning glory with the uninformed; but the chief and only solid advantage has been the pecuniary profits to those who, under this system, have been enabled to sell poor goods at a high price. One of the graduates of a dental college being asked by an examiner, "*What are the leading considerations or inducements for extracting the human teeth?* answered, "*I suppose, Sir, to make money*"! but where he got this lesson let others decide.

In conclusion, it seems to me to be a wide spread opinion among well informed professional men, that every student wishing to be an educated dentist, must study medicine thoroughly, and graduate as a medical man; but it by no means follows that we should expect all medical students to fit themselves for the speciality of the dentist, supposing a chair of dental surgery to exist in every medical school in the country. I am by no means for making dentists of all the doctors, but only for having medicine the basis of every dentist's education. Hence a professorship for dental surgery, attached to a medical college, need not necessarily embarrass or interfere with the chairs already established, and which are confessedly quite numerous enough. Those graduates who designed becoming dentists, would perhaps devote a third course to this branch, and it would rest with the professor who directed their studies and preparations for practice, to furnish the means of clinics, &c., in which to accomplish themselves in whatever is required. There are details in respect to which he who is called upon to fulfil the duties of a new chair, will of course find some difficulties at first; but they will be as readily overcome in medical, as they professedly have been in dental colleges.

